# Paving the way for precise diagnostics of antimicrobial resistant bacteria

**DOI:** 10.3389/fmolb.2022.976705

**Published:** 2022-08-12

**Authors:** Hao Wang, Chenhao Jia, Hongzhao Li, Rui Yin, Jiang Chen, Yan Li, Min Yue

**Affiliations:** ^1^ Institute of Preventive Veterinary Sciences & Department of Veterinary Medicine, Zhejiang University College of Animal Sciences, Hangzhou, China; ^2^ Hainan Institute of Zhejiang University, Sanya, China; ^3^ Department of Microbiology, Zhejiang Provincial Center for Disease Control and Prevention, Hangzhou, China; ^4^ Zhejiang Provincial Key Laboratory of Preventive Veterinary Medicine, Hangzhou, China; ^5^ State Key Laboratory for Diagnosis and Treatment of Infectious Diseases, National Clinical Research Center for Infectious Diseases, National Medical Center for Infectious Diseases, The First Affiliated Hospital, College of Medicine, Zhejiang University, Hangzhou, China

**Keywords:** antimicrobial resistance (AMR), AMR bacterial adaptation and evolutionary processes, advancements in biotechnology and computer science, reliable diagnostic tools, surveillance platform

## Abstract

The antimicrobial resistance (AMR) crisis from bacterial pathogens is frequently emerging and rapidly disseminated during the sustained antimicrobial exposure in human-dominated communities, posing a compelling threat as one of the biggest challenges in humans. The frequent incidences of some common but untreatable infections unfold the public health catastrophe that antimicrobial-resistant pathogens have outpaced the available countermeasures, now explicitly amplified during the COVID-19 pandemic. Nowadays, biotechnology and machine learning advancements help create more fundamental knowledge of distinct spatiotemporal dynamics in AMR bacterial adaptation and evolutionary processes. Integrated with reliable diagnostic tools and powerful analytic approaches, a collaborative and systematic surveillance platform with high accuracy and predictability should be established and implemented, which is not just for an effective controlling strategy on AMR but also for protecting the longevity of valuable antimicrobials currently and in the future.

## Introduction

The increasing prevalence of antimicrobial resistance (AMR) among bacterial pathogens poses one of the greatest threats to public health all over the world (Yue et al., 2021; Resistance, 2016), mainly because of the overuse of broad-spectrum antibiotics and potential antimicrobial-bacterium mismatch in empirical selections (Pan Hang et al., 2018; Jiang et al., 2019; Wang et al., 2019). Scientific reports indicate that death due to ineffective treatment of multidrug-resistant bacteria reaches over 100 cases daily in the European Union and the United States of America. Meanwhile, the increasing resistance to the “last-resort” antibiotics continuously concerns public health worldwide (Biswas et al., 2019; Elbediwi et al., 2019; Elbediwi et al., 2020).

To identify common AMR and guide diagnostics and effective treatment for bacterial infections, a gold-standard laboratory test used to determine the antimicrobial sensitivity of AMR organisms, termed antimicrobial susceptibility tests (AST), has been adopted in clinics for over 50 years. Traditional AST involves broth microdilution and disc diffusion on agar to determine the minimum inhibitory concentration (MIC). The MIC value indicates the lowest concentration of antibiotics where bacterial growth stops, and the growth level is usually measured by absorbance or light scattering. Before and during testing, multiple turnarounds of culturing the bacteria in the sample and isolating colonies and growth needed to be accomplished before AST. The whole pipeline takes at least 48 h, which usually provides the results after using an empirical prescription of antibiotics that might be misused. It has been estimated that around one-fifth of prescribed antibiotics from 2013 to 2015 were inappropriate in the United Kingdom (Smieszek et al., 2018). Also, currently, with over 500 million infected cases, COVID-19 has increased the setbacks in fighting against AMR (Knight et al., 2021; Ma et al., 2021). As many patients suffering from COVID-19 are being prescribed inappropriate antibiotics, this scenario might surge drug-resistant infections during the pandemic (Kariyawasam et al., 2022), particularly in developing countries where people lack antimicrobial stewardship programs and reliable diagnostics. Numerous AMR-associated outbreaks have been reported during the COVID-19 pandemic, such as carbapenemase-producing Enterobacteriaceae (Farfour et al., 2020; Tiri et al., 2020), highlighting the urgent demand for an efficient AMR surveillance system.

With decreasing sequencing costs and advancements in bioinformatics toolkits, whole-genome sequencing (WGS) for AMR diagnosing has been considered an efficient avenue for combating AMR (Schurch and van Schaik, 2017). The genotype-based alternative promises a quicker AMR detection than phenotypic AST by bypassing routine culturing for early detection and providing genomic insights into the mechanisms of AMR action, as well as critical clinical information, including pathogen species and virulence factors (Li et al., 2022). Utilizing the increasing WGS data and robust computerized analysis, machine learning has emerged to address the global AMR increase (Ren et al., 2021). It is a subfield of artificial intelligence focusing on developing algorithms to provide accurate and reliable predictions for clinical decisions. Conceptually, the genotype-based machine learning pipeline primarily requires the following steps: data selection (e.g., single-nucleotide polymorphisms and MIC value); encoding to be interpreted by algorithms; data training for model construction; evaluation and optimization by other dataset (Ren et al., 2021). The key challenge of these genotypic strategies that rely on the detection of AMR genes or SNPs to predict AMR phenotypes is the prevalent discrepancy between genotype and phenotype, which may produce detrimental effects on patients by prescribing inappropriate or unnecessary broad-spectrum antimicrobials (Yee et al., 2021). The phenotype of an organism is the observed expression of its genotype but is affected by multiple environmental variables (Davis et al., 2011; Rapun Mas et al., 2019; Urmi et al., 2020). The significance of the genotype–phenotype relationship emphasizes both their inherent correlation and the intricate uncertainty that should not be ignored but evaluated especially upon fighting AMR. Furthermore, AMR strains like *Pseudomonas aeruginosa* exhibit high plasticity of resistance phenotype driven by environmental changes (Dotsch et al., 2015). Overall, these phenomena suggest that bacteria may prefer different survival strategies under pressure circumstances. Various environmental signals, including antimicrobial and non-antimicrobial compounds, could significantly affect various susceptible phenotypes (Paudyal and Yue, (2019); Berendonk et al., 2015). AMR bacteria is an essential evolutionary outcome under selection pressure, and numerous unknown factors are involved, which implies limitations of single-dimension surveillance and diagnostic approaches. Therefore, more information on specific bacteria-host or -environment dynamics should also be considered to predict AMR.

The COVID-19 pandemic has seriously affected global efforts against AMR (Tomczyk et al., 2021), magnifying the insufficiency of available AMR surveillance capabilities. Accurate and rapid identification of antimicrobial resistance is still highly demanded, and a fine-tuned surveillance system remains the top priority of global public health. Since the culture-dependent traditional approaches cannot cope with the new challenge, various emerging novel methods have shown potential to solve the dilemma. Herein, we summarize these methods and propose an informed database/platform of resistant strains coordinated with reliable rapid molecular assays, multi-omics analysis, and machine learning to improve the performance of the established AMR surveillance system.

## Traditional phenotypical diagnostics

Traditional phenotypical diagnostics require isolating the initial bacterial colony from a clinical sample after incubation on a solid medium overnight. For the following broth microdilution assay, the monomicrobial cell is suspended under serial 2-fold dilutions with testing antibiotics in micro-wells. The lowest concentration of antibiotics in which bacteria cannot grow is recorded as the MIC value. For disc diffusion assay, the bacteria is spread onto an agar plate containing a fixed antibiotic concentration. After culturing overnight, the diameter of the circle zone of inhibition is measured. These two results are usually the basis for selecting effective antibiotics for bacterial infections. These traditional diagnoses of antimicrobial susceptibility of AMR strains have long relied on bacteria culturing, which is a time-consuming and labor-intensive process (Burnham et al., 2017). Moreover, the time lag of bacteria growth required for the conventional standard test may change the susceptibility to antimicrobials, particularly for important pathogens such as *Yersinia pestis* (Inglesby et al., 2000) and *Mycobacterium tuberculosis* (Votintseva et al., 2017). Although the culture-based antimicrobial susceptibility test used in clinical laboratories produces reproducible results, these assays *in vitro* cannot approximate the complicated interaction between microorganisms and the host in the tissue (Burnham et al., 2017), which may ultimately direct wrong drug selection (Wang et al., 2019; Elbediwi et al., 2021; Xu et al., 2021; Chen et al., 2022).

To tackle the dilemma of traditional phenotypical diagnostics, microbiologists have made novel efforts on establishing effective AST methodologies. Thore et al. (1977) found that ampicillin treatment induces a significant decrease of intracellular ATP, and an increase in inhibitory zone diameters, according to which they developed the luciferase assay-aided AST by measuring intracellular ATP of bacteria. For antifungal drug testing on *Candida albicans*, Pore established a flow cytofluorometric susceptibility test (FCST) by determining the cellular fluorescence intensity owing to antibiotic-caused membrane damage and the resulting uptake of propidium iodide or rose Bengal (Pore, 1990). To rapidly detect antibiotic resistance of *M. tuberculosis*, Riska et al. (1999) used phage with the luciferase reporter to infect the bacteria, and the causal detectable light indicates the infection quantitatively. Fredborg et al. (2013) introduced a real-time optical detection system to image bacterial growth and antimicrobial susceptibility. The automatically generated graphs supported by imaging material showed the antibiotic effects on bacteria, and it was able to screen 96 bacteria–antibiotic combinations at once. Idelevich et al. (2017) developed a methodology based on the BacterioScanTM216R laser scattering technology and following statistical analysis to rapidly discriminate the resistant and susceptible phenotypes of Gram-positive bacteria. Kang et al. (2019) built a droplet microfluidic device consisting of four integrated microdroplet arrays with each holding over 8,000 docking sites, which was capable of screening four antibiotics/pathogens simultaneously and assessing antibiotic sensitivity in 15–30 min. Greater flexibility can be achieved by operating microdroplet arrays individually or jointly. Combined with different aptamers, it has been divided into droplet microfluidics active microfluidics, paper microfluidics, and capillary-driven microfluidics (Han et al., 2021). But it has not been fully applied in clinical settings because of fabrication and operational complexity and less portability (Needs et al., 2020). More phenotypical diagnostics methods are summarized in Table 1.

**TABLE 1:** Brief summary of different phenotypic detection technologies.

Technology	Short description	Advantage	Disadvantage	References
Disk diffusion method with short incubation	Measure the inhibition zones after short-time incubation	Relatively rapid	A poorly controlled, unstandardized technic	Barry et al. (1973)
The firefly luciferase ATP assay	Growth of microorganisms is paralleled by an increase in ATP levels, and the level of ATP can be determined by the light produced by luciferase assay	Simple and highly sensitive	In many bacterial strains, accumulation of extracellular ATP may be prevented by the presence of ATP-ase activity	Thore et al. (1977)
Flow cytometry	Use flow cytometry to detect the membrane damage caused by drugs through increased cellular fluorescence	New and rapid; Improved sensitivity and standardization of the susceptibility test for the relatively large-celled fungi	PI and RB might be toxic to fungi upon binding to internal cell contents	Pore, (1990)
Luciferase Reporter Phage	When infected with mycobacteria, it will produce quantifiable light, the Bronx Box can detect the light	Rapid, reliable, inexpensive, simple, and low-tech manner	Lack of further validation	Riska et al. (1999)
Digital time-lapse microscopy	System introduces real-time tracking bacterial growth and antimicrobial susceptibility and generated graphs	The oCelloScope system is faster, portable, and requires low sample volumes to perform high-throughput bacterial susceptibility testing	The system is only suited for the imaging of fluid samples	Fredborg et al. (2013)
Microfluidic agarose channel system	Immobilize bacteria in microfluidic culture chamber, track single-cell growth by microscopy, and analyze the time lapse images of single bacterial cells to determine MICs	Fast and accurate	Low throughput and not friendly to use	Choi et al. (2013)
Forward Laser Scattering	Use narrow angle forward laser scattering to measure the light scattered from bacteria suspended in a liquid sample	The device is easy-to-use and has compact design and greatly shortens the time of AST	Unable to detect multiple resistance phenotypes	Idelevich et al. (2017)
MALDI-TOF MS direct-on-target microdroplet growth assay	Incubate the microorganisms on MALDI-TOF MS target and then detect the microorganisms grown by MALDI-TOF MS.	Rapid and accurate	This study uses only two different species	Idelevich et al. (2018)
Accelerate Pheno™ system	Transfer the BCB supernatant to a vial that is introduced into the device, then test automatically	Easy-to-use and fast	The number and characteristics of included samples in the study are limited	Descours et al. (2018)
Highly parallelized droplet microfluidic platform	Load water-in-oil droplets into four parallel arrays and then monitor the bacterial growth through the time-lapse imaging function	Fast and consumes less; screen four bacteria/drug combinations simultaneously	Only allow to detect the presence of a small proportion of resistant phenotypes; operation complexity	Kang et al. (2019)

## DNA-based detection

Offering speed and accuracy advantages compared with the traditional gold-standard phenotypic assay, molecular detection on the genetic determinants of resistance phenotype has been widely used in clinical settings. These DNA-based molecular assays can be primarily classified into three major platforms according to their underlying mechanics: polymerase chain reaction (PCR) or RT-PCR (Waseem et al., 2019), microfluidics-supported (Bai et al., 2022), and clustered regularly interspaced short palindromic repeats (CRISPR)-based methodologies (Kaminski et al., 2021). PCR has been extensively used today as a laboratory routine to identify bacteria from multiple environments and resistance genes (Rohde et al., 2017). Multiplex PCR, an optimized PCR by adding several primers, was described to detect nine clinically antibiotic resistance genes of *Staphylococcus aureus* in a single run including *mecA* (encoding methicillin resistance), *aacA*-*aphD* (aminoglycoside resistance), *tetK*, *tetM* (tetracycline resistance), *erm*(A), *erm*(C) (macrolide-lincosamide-streptogramin B resistance), *vat*(A), *vat*(B), and *vat*(C) (streptogramin A resistance) (Strommenger et al., 2003). With the high-throughput trait of microfluidic technology and rapid amplification at low temperature (37–42°C) of recombinase polymerase amplification (RPA), RPA-based microfluidics has been reported to identify *M. tuberculosis* by targeting 16 S rRNA with a sensitivity of 11 CFU/ml in 25 min (Tsaloglou et al., 2018); 10 copies of methicillin-resistant *S. aureus* DNA mixed with human whole blood were detected in 30 min by probing related SNP (Yeh et al., 2017); the major pathogenic bacteria in urinary tract infections *Escherichia coli*, *Proteus mirabilis*, *P. aeruginosa*, and *S. aureus* were successfully detected with detection limits of 100 CFU/ml from urine samples within 40 min by similar assay (Chen et al., 2018). The two leading platforms CRISPR-Cas13 based SHERLOCK (specific high-sensitivity enzymatic reporter unlocking) and CRISPR-Cas12 based DETECTR (DNA endonuclease-targeted CRISPR trans reporter) have realized that pathogen detections such as Zika and dengue viruses, bacterial isolates (*E. coli*; *Klebsiella pneumoniae*; *P. aeruginosa*; *M. tuberculosis*; *S. aureus*), AMR genes (*K. pneumoniae* carbapenemase and New Delhi metallo-beta-lactamase 1 (NDM-1), and even could detect cancer mutations with attomolar sensitivity and single-base mismatch specificity (Gootenberg et al., 2017; Chen et al., 2018). This CRISPR-based diagnostics pipeline involves pre-amplification of the target sequence, target recognizing, and cleaving by Cas nuclease, and result visualization. Once the target sequence is base-paired with the guide RNA of the CRISPR complex, the Cas protein cleaves the surrounding reporters (described as “collateral cleavage”), resulting in a detectable signal as a positive detection. CRISPR-Cas12 has already been used to diagnose *M. tuberculosis* with a sensitivity of two copies (Ai et al., 2019) and identify subspecies of the bacterium by targeting *rpoB* and erm genes (Xiao et al., 2020). Curti et al. (2020) successfully applied CRISPR-Cas12a to identify carbapenem-resistant genes such as *bla*_KPC_, *bla*_NDM_, and *bla*_OXA_ of *K. pneumoniae* at a pM level within 30 min. Shen et al. (2020) proposed a Cas13a-based system termed as APC-Cas by integrating an allosteric probe, and it was able to detect *Salmonella* in milk samples with single-cell sensitivity. A brief introduction of other different molecular assays is summarized in Table 2.

**TABLE 2:** A brief introduction of different molecular detection technologies.

Technology	Target	Description	Performance	References
Multicomponent nucleic acid enzyme-gold nanoparticle (MNAzyme-GNP) platform	Methicillin-resistant *Staphylococcus aureus*	Amplified target gene is chemically denatured and blocked to prevent rehybridization. When activated by blocked amplicons, MNAzyme cleaves the linker DNA, rendering GNPs monodispersed. In the absence of the target gene, the linker DNA remains intact owing to inactive MNAzyme and causes GNPs to aggregate	100 DNA copies/μL	Abdou Mohamed et al. (2021)
CRISPR-Cas9 triggered two-Step Isothermal amplification method	*Escherichia coli* O 157:H7	The target virulence gene sequences are recognized and cleaved by the CRISPR-Cas9 sy (Sun et al., 2020) stem and trigger strand displacement amplification and rolling circle amplification	4 CFU/ml	Sun et al. (2020)
A clustered regularly interspaced short palindromic repeat (CRISPR)-mediated surface-enhanced Raman scattering (SERS) assay	*S. aureus*, *Acinetobacter baumannii*, and *Klebsiella pneumoniae* with multidrug-resistance	The Au MNP-dCas9/gRNA probe and genomic DNA mixed in a single reaction tube. Next, methylene blue (MB) is added to the tube. Finally, the MDR bacterial gene-bound Au MNP-dCas9/gRNA probe is collected with the assistance of an external magnet, and the SERS measurement is accomplished	fM level	Kim et al. (2020)
FLASH (Finding Low Abundance Sequences by Hybridization)	*S. aureus* with antimicrobial resistance	Combines CRISPR/Cas9 and multiplex PCR	35 copies	Quan et al. (2019)
A paper-based chip integrated with loop-mediated isothermal amplification (LAMP) and the “light switch” molecule [Ru (phen)2dppz]2+	Methicillin resistant *S. aureus*, *E. coli*, *Listeria Monocytogenes*, and *Salmonella*	The amplification reagents can be embedded into test spots of the chip in advance, thus simplifying the detection procedure. [Ru (phen)2dppz]2+ was applied to intercalate into amplicons for product analysis, enabling this assay to be operated in a wash-free format	100 copies/μL	Li et al. (2018)
Droplet Digital PCR	Clarithromycin-resistant *Helicobacter pylori*	A method to simultaneously quantify *H. pylori* clarithromycin-resistant (mutant) and -susceptible (wild-type) 23 S rRNA gene alleles in both stomach and stool samples using droplet digital PCR.	Discriminate the clarithromycin resistance strain DNAs (A2143G, A2142G, and A2142C) mixed with the wild-type strain at ratio of 0:1, 1:100, 1:10, 1:1, 10:1, 100:1, and 1:0	Sun et al. (2018)
Digital real-time loop-mediated isothermal amplification (dLAMP) assay	*E. coli*	AST results can be obtained by using digital nucleic acid quantification to measure the phenotypic response of samples exposed to an antibiotic for 15 min	Ultrafast (7 min)	Schoepp et al. (2017)

WGS emerges as a powerful tool for understanding the genetic makeup of bacteria AMR. As the high-throughput sequencing technology developed in the mid-2000 s, it can illustrate a landscape of the whole resistome in a couple of days with a sophisticated downstream bioinformatic pipeline (Chan, 2016; Goodwin et al., 2016). The new advancement in sequencing technology shortens the time of the whole procedure from days to a few hours (Shendure et al., 2017). According to these known genetic determinants, mass software tools have been designed to detect and predict drug resistance (Henry et al., 2014; Sallet et al., 2019). An immense amount of sequencing data generated annually is used to build a global genotype–phenotype database contributing to a worldwide surveillance system on the AMR strains (Gardy and Loman, 2017). Once the public-health-threatening “criminal” is identified by fingerprinting with a genetic test, its AMR profile will be provided for treatment by the database in time. The Antibiotic Resistance Monitoring, Analysis, and Diagnostics Alliance (https://joinarmada.org/), a nonprofit global organization combatting superbugs with a database of bacterial genomes, has been established to create a specific “criminal database” of AMR pathogens, collecting an unprecedented amount of bacterial strains and details of their antibiotic resistance profiles, genetic identity and epidemiology from a global network of hospitals, veterinarians, scientists, and other advocates. Alcock et al. (2020) developed the Comprehensive Antibiotic Resistance Database providing data of curated reference sequences and SNPs, models, and algorithms, helping users with genotype analysis and phenotype prediction of AMR. The notable AMR databases also include ResFinder (Zankari et al., 2012), ARG-ANNOT (Gupta et al., 2014), the and National Center for Biotechnology Information Pathogen Detection Reference Gene catalog (Sayers et al., 2022). These primary AMR databases curate information from the scientific research into the collection for further sequence analysis and knowledge integration. WGS has also been applied for the risk assessment of probiotic lactic acid bacteria (Peng et al., 2022). However, to detect the genetic determinants of AMR, knowledge or prior research regarding which gene is responsible for the resistance is required for the method before diagnostics. Since complex features and mechanisms of AMR remain obscure, the genotype–phenotype method seems less valuable than expected for AMR identification (Wu et al., 2021; Hu et al., 2022).

## Machine learning facilitates the underappreciated antimicrobial resistance prediction

Learning essential characteristics directly from the data of genomic sequences, machine learning obviates the need for prior knowledge of resistant strains caused by unknown mechanisms. It has been proven valuable for predicting AMR with an unbiased method (Martens et al., 2016; Her and Wu, 2018; Wheeler et al., 2018). Unlike direct detection of AMR genes, machine learning often takes more extensive features indiscriminately into AMR identification, such as SNP, K-mer, and pan-genome (Her and Wu, 2018; Nguyen et al., 2019; Ren et al., 2021), and even some parameters affecting the performance and reliability of machine learning-based antibiotic susceptibility tests need to be evaluated (Hicks et al., 2019). Different from the traditional DNA alignment approach, machine learning is mathematically oriented by algorithms including logistic regression (LR), support vector machine (SVM), random forest (RF), and convolutional neural network (CNN) (Her and Wu, 2018). In order to confirm the WGS data to the valid format of existing classification algorithms, these data are usually encoded before analysis by the algorithms as mentioned above for model construction, and standard encoding methods include Label, One-Hot, and frequency matrix chaos game representation (FCGR), etc., (Her and Wu, 2018). The final prediction model will be tested and evaluated by other datasets, and the model established might vary for different coding methods and algorithms/classifiers involved. Leveraged with big data from WGS or other NGS technologies, machine learning facilitates potential AMR prediction and helps direct informed drug decisions (Moradigaravand et al., 2018; Ren et al., 2021). Recent literature in the field is presented in Table 3.

**TABLE 3:** Recent progresses on ML for AMR prediction.

Strain	Extracted feature	Algorithm	Predicting target	Result	References
*E. coli*	Pan-genome	Support Vector Machine; Naïve Bayes (NB); Adaboost; Random Forest	Meropenem; gentamicin; ciprofloxacin; trimethoprim/sulfa; ethoxazole ampicillin; cefazolin; ampicillin/sulbactam; ceftazidime; cefepime; piperacillin/tazobactam; tobramycin; ceftriaxone	Support Vector Machinen for 12 drugs’ AUC: 0.67–0.82	Her and Wu, (2018)
				Naïve Bayes (NB) for 12 drugs’ AUC:0.69–0.85	
				Adaboost for 12 drugs’ AUC: 0.54–0.86	
				Random Forest for 12 drugs’ AUC: 0.51–0.82	
Nontyphoidal *Salmonella*	k-mer	XGBoost	Ampicillin; amoxicillin-clavulanic acid; ceftriaxone; azithromycin; chloramphenicol; ciprofloxacin; trimethoprim-sulfamethoxazole; sulfisoxazole; cefoxitin; gentamicin; kanamycin; nalidixic acid; streptomycin; tetracycline; ceftiofur	XGBoost for 12 drugs’ Accuracy: 0.33–0.91	Nguyen et al. (2019)
*E. coli*	SNP	Support Vector Machine; Logistic Regression; Random Forest; Convolutional Neural Network	Ciprofloxacin; cefotaxime; ceftazidime; gentamicin	Support Vector Machine for 4 drugs’ Accuracy: 0.75–0.88	Ren et al. (2021)
				Logistic Regression for four drugs’ Accuracy: 0.77–0.85	
				Random Forest for 4 drugs’ Accuracy: 0.77–0.92	
				Convolutional Neural Network for four drugs’ Accuracy: 0.71–0.84	
*K. pneumoniae; A. baumannii*	k-mer	Machine Classification; Random Forest; Random Forest Regression	Ciprofloxacin; azithromycin	Three model’s Accuracy for CIP: ≥0.93; Three model’s Accuracy for AZI: 0.57–0.94	Hicks et al. (2019)
*Bacitracin Vancomycin*	Protein sequences	Support Vector Machine	AMR or non-AMR	Classification accuracies 87%–90%	Chowdhury et al. (2020)
*Neisseria gonorrhoeae*	pan-genome	Logistic Regression; Random Forest; Gradient Boosting Decision Tree; Support Vector Machine	Penicillin; tetracycline; cefixime; ciprofloxacin; azithromycin	AUC and Recall values of the training and testing datasets were >0.80 in at least one machine learning model for all antibiotics	Li et al. (2020)

Clinical application of machine learning for AMR diagnosing, preventing, and understanding still stands in the infancy stage because of the complexity of AMR itself and the deployment of machine learning for clinical practice. That genomic-based machine learning aiming to predict the MIC value is the foremost strategy for AMR prediction (Coelho et al., 2013; Pesesky et al., 2016; Nguyen et al., 2019). But challenges/limitations remain to be overcome. Training data, (e.g., MIC value) might not be available for all laboratories and sections, which would affect the clinical effectiveness of the model, which suggests unified standardization of operating protocols or guidelines is highly demanded. For algorithms used in the model, different ones reach different outcomes, indicating a comparative study of algorithms should be conducted before applying for better accuracy. In addition, the prediction result should be understandable to intended users. For the breadth of machine learning prediction, studies have primarily predicted the most common antibiotics used on a few common bacterial pathogens, but the clinical encountered pathogens far outrange the prediction covering. Special effort should also be taken on the highly clinically significant organisms, including vancomycin-non-susceptible *S. aureus* (Shariati et al., 2020) and the ones with AMR phenotypic plasticity, including *P. aeruginosa* (Khaledi et al., 2020). Furthermore, data on AMR to new antibiotics (e.g., eravacycline, ceftazidime-avibactam, and cefiderocol) are also required to examine in clinical settings. With the exponential increase of available biological data, massive investments in computational power, critical advancements in algorithm performance, and increasing global involvement worldwide, machine learning will accelerate the clinical diagnosis of AMR to a new level.

## Transcriptomic and proteomic approaches

The DNA-based method only detects the existence of these potential resistance DNA traits without providing information regarding efficient transcription, expression, and antimicrobial susceptibility. The feedback in bacterial transcriptome and proteome to antibiotics exposure offers real-time dynamic information of relevant genes. Their expression change in mRNA and protein levels is endowed with key diagnostic values for AMR detection. Some efforts have also been made in transcriptome and proteome, but remain limited. Steinberger-Levy et al. (2016) established a research model by exposing 53 strains of *Y. pestis* to the growth-inhibiting concentrations of ciprofloxacin for different time periods and investigates the expression changes of specific marker genes by transcriptomic DNA microarray analysis. Eleven transcripts with significant change were identified as the potential biomarkers, of which four mRNAs (*recA*, *pla2*, *recN*, and *dinI*) were selected to determine the susceptibility by performing quantitative RT-PCR, and the results are consistent with MIC values. Rohde et al. (2016) used fluorescence *in situ* hybridization and immunofluorescence tests to locate and quantify the mRNA and protein existence of the TEM β-lactamase, conferring ampicillin resistance in the *E. coli*. Chen et al. (2020) performed a shotgun proteomics assay and WGS on four isolates of *Campylobacter jejuni* and analyzed the data in the Comprehensive Antibiotic Resistance Database for AMR detection. It was found that both genomic and proteomic approaches can identify molecular determinants responsible for resistance to tetracycline and ciprofloxacin, in line with their phenotypes. These methods require several steps and analyses, which are more suitable for basic research, but not feasible for the field application. For rapid RNA detection, the RNA-targeting CRISPR-associated enzyme Cas13a directly binds to the target RNA and releases the positive signal (Shinoda et al., 2021). Combined with RT-RPA that transforms the RNA to cDNA and amplifies DNA with primers, the DNA-targeting enzyme Cas12a can also be applied to the RNA detections (Malci et al., 2022). In clinical practice, rapid molecular diagnostics help distinguish viral infections from bacterial infections, preventing unnecessary treatment of antibiotics. Only molecular-based methods cannot differentiate which bacteria contain the AMR element (plasmids containing AMR gene) in the mixed bacterial infection of clinical use. If the carrying AMR genes among various bacterial populations can be identified specifically, we might use a more targeting or effective strategy against AMR increase, suggesting this field might need further investigation.

## Current challenge

**FIGURE 1:**
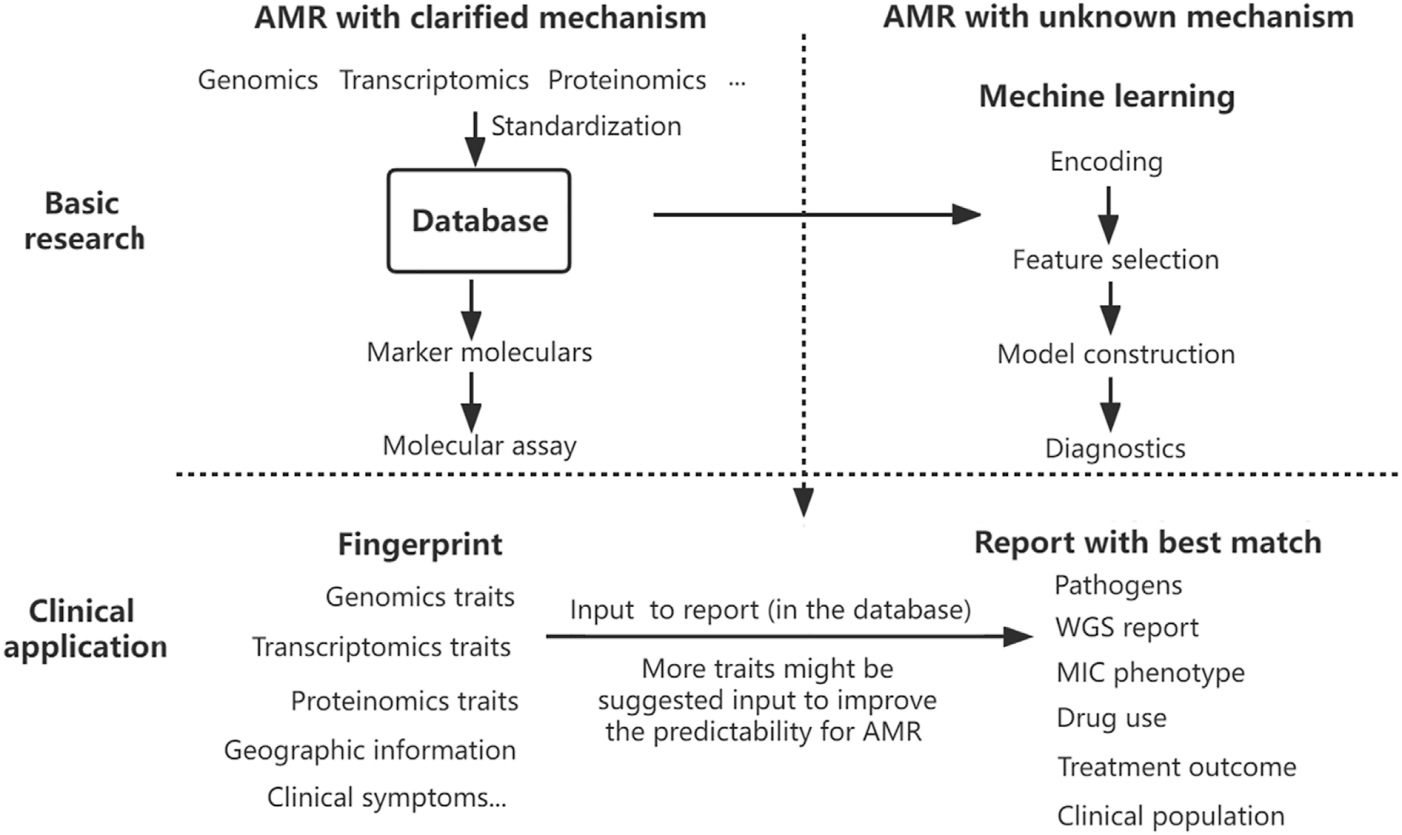
Proposed AMR surveillance model. Various data on antibiotic resistance have been collected into the database, which provides marker molecules to detect AMR with clarified mechanism and fuels the model training of machine learning to predict the AMR with an unknown mechanism. The results of rapid molecular detection on multi-traits and other information help fingerprint the pathogens, and the database interface will report the best matched or advised treatment decision directly. Taking advantage of the rapidity of molecular assays and the precision of machine learning fueled by a constant flow of multi-dimensional data, the AMR surveillance platform optimizes drug selection, antimicrobial stewardship, and epidemiological monitoring.

A comprehensive analysis has suggested more factors to make more precision diagnostics and informed preclinical decisions (Sommer et al., 2017). To deal with this compiling problem, a full-scale assessment system should be set up to predict AMR precisely. Establishing a database on genotype–phenotype data is a good start, but a more comprehensive platform of AMR strain is highly needed for the precision diagnostics of AMR. Utilizing the multi-dimension data collected from the genome, transcriptome, and proteome profiles of AMR bacteria isolated from hospitals, doctor offices, clinical laboratories, communities, and veterinary sources, a ‘criminal database’ of AMR pathogens can be geographically and phylogenetically established. After fingerprinting those known genetic makers by rapid molecular test and sampling information, the database will report with the best match containing current and historical essential details, including WGS report, pathogen, MIC phenotype, drug use and treatment outcomes, clinical population, and other accessory information. With the data collected from multiple sources, bacterial AMR patterns will be well analyzed by machine learning, enabling reliable digital models of a particular drug, pathogen, and clinical population. Machine learning will act as an alternative to pinpoint that unknown resistance. Surveillance and diagnostic interaction significantly improve antimicrobial selection and epidemiological monitoring in the pipeline. Once database standardization is well-established, this constantly updated global detection platform combining diagnostics, surveillance, and prediction of AMR would improve proactive identification and mitigate the emerging crisis of AMR (Figure 1).

From the clinical perspective, the priority is confirming whether antimicrobial therapies are needed in a particular case; the second is which drug is suitable for an optimized treatment if a bacterial infection is already present. Apparently, the most clinical urgency for patients and physicians is not knowing which bacteria is resistant to what antimicrobial but determining which narrow-spectrum drug can eliminate the pathogen. Most diagnostic methods aim to identify drug-resistant determinants of superbugs, but few studies explored if there are any drug-susceptible determinants or other multi-omics traits in AMR strains. These drug-susceptible feature data can be used for model training in machine learning, and molecular-based assays can detect drug-susceptible markers directly. More innovative efforts are needed in this area in the future.

## Future direction

In the coming decade, advancements in technical and computational tools for multi-omics approaches are continually improving knowledge regarding the diverse AMR mechanisms and integrating a deep mechanistic understanding of AMR determinants with a broader systematic analysis of microorganisms. The ultimate goal will lead to a revolutionary change in AMR diagnostics, significantly guiding the surveillance of AMR threats and finally slowing down the rising crisis.
